# Relationship Between Body Mass Index at Age 3 Years and Body Composition at Age 11 Years Among Japanese Children: The Shizuoka Population-Based Study

**DOI:** 10.2188/jea.JE20110113

**Published:** 2012-09-05

**Authors:** Katsuyasu Kouda, Harunobu Nakamura, Yuki Fujita, Masayuki Iki

**Affiliations:** 1 Department of Public Health, Kinki University Faculty of Medicine, Osaka-Sayama, Osaka, Japan; 1 近畿大学医学部公衆衛生学教室; 2 Department of Health Promotion and Education, Graduate School of Human Development and Environment, Kobe University, Kobe, Japan; 2 神戸大学大学院人間発達環境学研究科健康発達論

**Keywords:** body composition, child, development, nutrition

## Abstract

**Background:**

A few studies reported an association between body weight during early childhood and body composition in later life, as measured by dual-energy X-ray absorptiometry (DXA); however, none of those studies investigated an East Asian population. In a Japanese population, we examined the association between body weight at age 3 years and body composition at age 11 years, as measured using DXA.

**Methods:**

The source population was 726 fifth-grade school children enrolled at 3 public schools in Shizuoka Prefecture, Japan from 2008–2010. All children who lived in the study area went to 1 of these 3 schools. DXA was used to obtain data on body composition, and the Maternal and Child Health Handbook was used to calculate body mass index (BMI). The general linear model was used for statistical analysis.

**Results:**

We were able to analyze data on body composition at age 11 years and BMI in early childhood for 550 children. BMI at age 3 and change in BMI z-score from birth to age 3 were positively associated with bone mineral content (BMC), fat-free soft tissue mass (FFSTM), and fat mass (FM) at age 11. After adjusting for confounding factors, mean BMC, FFSTM, and FM were significantly lower among children who were underweight at age 3 and significantly higher among children who were overweight at age 3, as compared with values for normal-weight children at age 3.

**Conclusions:**

Among Japanese children, body weight at age 3 years predicts body composition at age 11 years.

## Abstract (ja)

**【背景】:**

東アジアの集団において、幼児期の体格とその後の体組成の関連についての詳細な研究はない。今回は日本人集団における3歳時の体格と11歳時の体組成の関連について、二重エネルギーエックス線吸収法（DXA法）を用いて検討した。

**【方法】:**

調査対象集団は2008年から2010年に静岡県下の3つの公立小学校に在籍した726名の児童である。体組成はDXA法で測定し、幼児期の体格（BMI）は母子健康手帳から入手した。統計解析には一般線形モデルを用いた。

**【結果】:**

調査対象集団のうち550名から体組成と幼児期の体格のデータを得た。3歳時のBMIは11歳時の全身骨塩量（BMC）、全身除脂肪軟部組織量（FFSTM）、全身脂肪量（FM）と正の関連を示した。出生から3歳のBMI z-scoreの変化も11歳時のBMC、FFSTM、FMと正の関連を示した。交絡因子調整後の11歳時のBMC、FFSTM、FMの平均値は、共に3歳時に低体重であった者が正常体重よりも低く、3歳時に過体重であった者は正常体重よりも高かった。

**【結論】:**

3歳時の体格はその後の体組成を予測した。

## INTRODUCTION

Many studies have suggested that there are several critical periods in obesity development.^1^^–^^4^ A recent prospective cohort study found that an increase in weight velocity from age 1 to 5 years is the most important predictor of body mass index (BMI) in young adults.^5^ Another, longitudinal study found that most excess weight before puberty is gained before age 5 years and that weight at this age closely predicts weight at puberty.^6^ One hypothesis attracting increasing attention is that childhood obesity can be predicted by body weight in infancy and during preschool. However, the relevant studies used body weight during later life as an outcome. Because body weight reflects both lean and fat mass (FM), it is an insufficient index of obesity, which is defined as abnormal or excessive fat accumulation.^7^

Pubertal body composition is believed to be important due to its linkage with the risk of chronic diseases in adulthood and its usefulness for disease risk assessment.^8^ Accordingly, body composition in pubertal children needs to be assessed as an outcome. A prospective cohort study using foot–foot impedance reported that rapid weight gain from age 1 to 4 years is associated with FM and lean mass at age 9 years.^9^ In addition, a study using air-displacement plethysmography revealed an association between weight gain at age 3 to 6 years and body composition at age 17 years.^10^

Dual-energy X-ray absorptiometry (DXA) can be used to evaluate childhood obesity and determine FM, fat-free soft tissue mass (FFSTM), and bone mineral content (BMC).^11^ DXA is increasingly being used as a criterion or reference for comparison with other measurements of body composition.^12^ Only a few studies have examined the association between body weight during early childhood and body composition, as measured by DXA, in later life.^13^^–^^17^ In 1 such study in southwest England, larger body size at birth was associated with higher fat and higher lean mass at age 9 to 10 years.^13^ The Fels Longitudinal Study reported that white infants with catch-up growth tended to have greater FM in adulthood.^14^ In addition, a study in Greater London and Cambridgeshire reported that higher birth weight was associated with higher fat-free mass in later life.^16^ The same study group also reported an association between postnatal weight gain and body composition in obese whites aged 5 to 22 years.^17^ Although these findings suggest that body weight in early childhood might be associated with pubertal body composition in the Japanese population, the relationship between body fat and BMI is known to differ across ethnic groups.^18^ A World Health Organization Expert Consultation reported that Asians have a higher percentage of body fat than do whites with the same BMI.^19^ Nevertheless, there are no data for East Asian populations on the association between body mass in early childhood and body composition in later life.

In Japan, maternal and child health services requires that 3-year-old children undergo physical examinations, and these are routinely performed by local governments for all children.^20^ Because pubertal body composition is linked with onset of chronic diseases in adulthood,^8^ knowledge of a relationship between BMI at age 3 years (as determined using data from health examinations of 3-year-olds) and pubertal body composition may be useful for early childhood prevention of chronic disease in adulthood. Accordingly, we investigated the association between body weight during early childhood (age 0–3 years) and body composition at age 11 years, as measured by DXA, in a Japanese population.

## METHODS

### Study population

The source population was fifth-grade school children who attended Aritama Elementary School, Fukuroi-kita Elementary School, and Sekishi Elementary School in Shizuoka Prefecture, Japan (726 children: 393 boys and 333 girls). These are public schools in Hamamatsu City and Fukuroi City. All children who lived in the study area went to 1 of these 3 schools, as there are no private schools in these regions. Our study was approved by the Ethics Committee of Kinki University Faculty of Medicine and was performed in accordance with the ethical standards set forth in the 1964 Declaration of Helsinki. The study procedures were explained to all parents of participants, and written informed consent was obtained from the parents.

### Body composition

Whole-body and regional compositions (BMC, FFSTM, and FM) were determined with a single DXA scanner (QDR-4500A, Hologic Inc., Bedford, MA, USA) that was brought to the schools in a mobile test room. Testing was performed at Aritama Elementary School (Feb 2008, Feb 2009, Feb 2010, and Nov 2010), Fukuroi-kita Elementary School (Sep 2009), and Sekishi Elementary School (Dec 2010). Children wore light clothing without metal objects while undergoing whole-body scanning. Truncal FM was distinguished from arm, leg, and head FM, as previously described.^21^ Arm, leg, and head measurements were isolated from trunk measurements using computer-generated default lines, with manual adjustment, on the anterior view planogram. Specific anatomical landmarks (chin, center of the glenohumeral joint, and femoral neck axis) defined the trunk.

Body composition components were evaluated using height-normalized indices to avoid the ambiguities that result when these components are reported as percentages of body weight.^22^ BMC index (BMCI, kg/m^2^), FFSTM index (FFSTMI, kg/m^2^), and FM index (FMI, kg/m^2^) were calculated as BMC, FFSTM, and FM weight (kg) divided by the height squared (m^2^), respectively.

### Anthropometry

Using the Maternal and Child Health Handbook (MCH Handbook), parents transcribed body weight and length at birth to a questionnaire. The MCH Handbook system in Japan is designed to promote maternal/child health among all pregnant women and children younger than 6 years.^23^ The MCH Handbook is distributed by local governments when pregnant women register, and almost 100% of pregnant women use the handbook.^24^ In Japan, physical examinations of 3-year-old children are required by maternal and child health services and are routinely performed by local governments for all children.^20^ Height and weight, among other variables, are measured during this examination.^25^ A record of the health examination at age 3 (including body weight and height) is also included in the MCH Handbook.^24^ Body weight and height at age 3 were transcribed to the questionnaire from the Maternal MCH Handbook by parents. Body weight and height of the mother of children at age 11 years were also obtained by self-report. Body weight, height, and waist circumference (WC) at age 11 years were measured at the time of body composition measurements. The method for measuring WC was previously reported in detail.^21^ In brief, horizontal WC was measured at the umbilicus while standing, after normal expiration.

BMI was calculated as weight (kg) divided by the height (or newborn length) squared (m^2^). The international BMI cut offs for child overweight at age 3 years (based on the adult cut off of BMI 25 kg/m^2^) were used.^26^ Similarly, the international BMI cut offs for child underweight at age 3 (based on the adult cut off of BMI 18.5 kg/m^2^) were used.^27^ Age- and sex-specific BMI z-scores were calculated as set forth by the World Health Organization Multicentre Growth Reference Study Group.^28^

### Statistical analysis

The unpaired *t*-test was used to compare characteristics of boys and girls. Pearson’s correlation test was used to assess correlations of body composition components at age 11 with BMI in early childhood and BMI of mothers of 11-year-old children. Mean values for components of body composition were calculated after adjusting for potential confounding factors, including sex, BMI at birth, and maternal BMI, using the general linear model with multiple comparisons. A *P*-value less than 0.05 was considered statistically significant. Statistical analyses were performed with SPSS Statistics Desktop for Japan, Version 20.0 (IBM Japan, Ltd., Tokyo, Japan).

## RESULTS

Among the source population, 637 children (336 boys and 301 girls) underwent DXA measurement of body composition. From these 637 children, we were able to obtain information on BMI at birth, BMI at age 3 years, and current maternal BMI for 550 (286 boys and 264 girls; mean age, 11.2 years; 75.8% of the source population). The data from these 550 children were analyzed.

Table 1 shows the characteristics of the participants. At birth and age 3, height and weight were significantly higher in boys than in girls. At age 11 years, height, BMC, and total FM was significantly higher in girls than in boys, and FFSTMI was significantly lower in girls than in boys.

**Table 1.** Characteristics of participants

	Boys (*n* = 286)	Girls (*n* = 264)	*P*-value^a^
At birth			
Length (cm)	49.28 ± 0.13	48.67 ± 0.14	0.001
Weight (kg)	3.04 ± 0.02	2.92 ± 0.02	<0.001
BMI (kg/m^2^)	12.47 ± 0.07	12.28 ± 0.07	0.08
At age 3 years			
Height (cm)	95.38 ± 0.30	94.21 ± 0.25	0.003
Weight (kg)	14.39 ± 0.10	13.92 ± 0.10	0.001
BMI (kg/m^2^)	15.79 ± 0.07	15.67 ± 0.08	0.3
At age 11 years			
Height (cm)	141.86 ± 0.39	144.15 ± 0.39	<0.001
Weight (kg)	34.67 ± 0.42	35.20 ± 0.40	0.4
BMI (kg/m^2^)	17.10 ± 0.15	16.83 ± 0.14	0.2
WC (cm)	63.66 ± 0.42	63.54 ± 0.37	0.8
SBP (mm Hg)	102.55 ± 0.63	105.25 ± 0.68	0.004
DBP (mm Hg)	55.33 ± 0.46	57.95 ± 0.50	<0.001
BMC (kg)	1.02 ± 0.01	1.06 ± 0.01	0.01
BMCI (kg/m^2^)	0.51 ± 0.00	0.51 ± 0.00	0.8
FFSTM (kg)	28.00 ± 0.25	27.91 ± 0.25	0.8
FFSTMI (kg/m^2^)	13.84 ± 0.07	13.36 ± 0.07	<0.001
Trunk FM (kg)	2.14 ± 0.09	2.38 ± 0.08	0.054
Trunk FMI (kg/m^2^)	1.04 ± 0.04	1.13 ± 0.04	0.1
Total FM (kg)	6.98 ± 0.21	7.61 ± 0.18	0.03
Total FMI (kg/m^2^)	3.42 ± 0.09	3.63 ± 0.08	0.1

BMI, body mass index; WC, waist circumference; SBP, systolic blood pressure; DBP, diastolic blood pressure; BMC, bone mineral content; BMCI, bone mineral content index; FFSTM, fat-free soft tissue mass; FFSTMI, fat-free soft tissue mass index; FM, fat mass; FMI, fat mass index.

Values represent mean ± standard error.

^a^The unpaired *t*-test was used to compare characteristics of boys and girls.

Table 2 shows the correlations of body composition components at age 11 with early childhood BMI and maternal BMI. BMI at age 3 was significantly correlated with BMCI, FFSTMI, trunk FMI, and total FMI at age 11 in both sexes. In addition, change in BMI z-score from birth to age 3 was significantly correlated with BMCI, FFSTMI, trunk FMI, and total FMI at age 11 in both sexes. BMI at birth was weakly correlated with FFSTMI in boys, but it was not correlated with BMCI, FFSTMI, trunk FMI, or total FMI in girls. BMI among mothers of children aged 11 years was significantly correlated with FFSTMI, trunk FMI, and total FMI of children at age 11 in both sexes.

**Table 2.** Correlations of body composition components at age 11 years with early childhood BMI and maternal BMI

	BMI at birth		BMI at age 3 years		Change in BMI z-score		Maternal BMI	
				
	*r*	*P*	*r*	*P*	*r*	*P*	*r*	*P*
Boys (*n* = 286)								
BMC (kg)	0.130	0.03	0.280	<0.001	0.108	0.07	0.107	0.07
BMCI (kg/m^2^)	0.013	0.8	0.270	<0.001	0.194	<0.001	0.083	0.2
FFSTM (kg)	0.211	<0.001	0.291	<0.001	0.053	0.4	0.175	0.003
FFSTMI (kg/m^2^)	0.143	0.02	0.338	<0.001	0.142	0.02	0.208	<0.001
Trunk FM (kg)	0.060	0.3	0.221	<0.001	0.120	0.04	0.306	<0.001
Trunk FMI (kg/m^2^)	0.044	0.5	0.220	<0.001	0.131	0.03	0.312	<0.001
Total FM (kg)	0.094	0.1	0.267	<0.001	0.127	0.03	0.296	<0.001
Total FMI (kg/m^2^)	0.068	0.3	0.264	<0.001	0.145	0.01	0.302	<0.001

Girls (*n* = 264)								
BMC (kg)	0.073	0.2	0.186	0.002	0.095	0.1	0.100	0.1
BMCI (kg/m^2^)	0.057	0.4	0.230	<0.001	0.143	0.02	0.090	0.1
FFSTM (kg)	0.096	0.1	0.231	<0.001	0.113	0.07	0.158	0.01
FFSTMI (kg/m^2^)	0.088	0.2	0.329	<0.001	0.200	0.001	0.184	0.003
Trunk FM (kg)	0.078	0.2	0.240	<0.001	0.135	0.03	0.253	<0.001
Trunk FMI (kg/m^2^)	0.066	0.3	0.251	<0.001	0.153	0.01	0.265	<0.001
Total FM (kg)	0.099	0.1	0.290	<0.001	0.159	0.01	0.285	<0.001
Total FMI (kg/m^2^)	0.087	0.2	0.310	<0.001	0.185	0.003	0.300	<0.001

BMI, body mass index; BMC, bone mineral content; BMCI, bone mineral content index; FFSTM, fat-free soft tissue mass; FFSTMI, fat-free soft tissue mass index; FM, fat mass; FMI, fat mass index.

Change in BMI z-score was calculated using BMI z-score at birth and BMI z-score at age 3 years.

Pearson’s correlation test was used.

Table 3 shows adjusted mean BMCI, FFSTMI, and total FMI at age 11. After adjusting for confounding factors (including sex, BMI at birth, and maternal BMI), BMCI, FFSTMI, and FMI were significantly lower in children who were underweight at age 3 and significantly higher in children who were overweight at age 3, as compared with values for normal-weight children. There were also significant dose-dependent trends for body composition at age 11, relative to weight status at age 3.

**Table 3.** Adjusted mean values for body composition components at age 11 years by weight status at age 3 years

	BMI at age 3 years			*P* for trend
	
	Underweight (*n* = 68)	Normal-weight (*n* = 454)	Overweight (*n* = 28)	
BMCI (kg/m^2^)	0.48 ± 0.01^a^	0.51 ± 0.00	0.54 ± 0.01^b^	<0.001
FFSTMI (kg/m^2^)	13.08 ± 0.14^a^	13.63 ± 0.05	14.56 ± 0.21^a^	<0.001
Total FMI (kg/m^2^)	2.95 ± 0.17^c^	3.52 ± 0.06	4.88 ± 0.25^a^	<0.001

BMI, body mass index; BMCI, bone mineral content index; FFSTMI, fat-free soft tissue mass index; FMI, fat mass index.

Values are mean ± standard error adjusted for sex, BMI at birth, and maternal BMI.

^a^Compared with the normal group (*P* < 0.001) using the general linear model.

^b^Compared with the normal group (*P* = 0.009) using the general linear model.

^c^Compared with the normal group (*P* = 0.001) using the general linear model.

## DISCUSSION

This is the first study to report an association between BMI in early childhood and body composition in later childhood in a Japanese population. In the present study, BMI at age 3 years was positively associated with body composition components at age 11 years. Specifically, as compared with normal-weight children at age 3, BMC, fat-free mass, and FM were lower in 11-year-old children who were underweight at age 3 and higher in children who were overweight at age 3.

Few studies have examined the association between body weight during early childhood and body composition, as measured by DXA, in later life.^13^^–^^17^ In the large-scale Avon Longitudinal Study of Parents and Children (3006 boys and 3080 girls), conducted in southwest England, a higher ponderal index at birth was associated with both higher fat mass and higher lean mass at age 9 to 10 years.^13^ In a relatively small-scale study in Greater London and Cambridgeshire, United Kingdom, Chomtho et al reported that birth weight was positively associated with fat-free mass, but not with FM, later in life.^16^ In the same geographic region, Wells et al reported that birth weight was only a weak predictor of tissue mass.^17^ In the present Japanese population, although there was a very weak association between BMI at birth and lean mass in boys, BMI at birth was not significantly associated with body fat later in life in either sex. Thus, it might be difficult to predict late body composition by using BMI at birth. With respect to growth patterns in early childhood, Chomtho et al reported that relative weight gains from 0 to 3 months and from 3 to 6 months were positively associated with childhood FM and fat-free mass in Greater London and Cambridgeshire.^15^ The Fels Longitudinal Study also reported that infants with catch-up growth tended to have greater FM in adulthood than did infants with catch-down growth or no significant change in weight status in a white population.^14^ In the present Japanese population, BMI at age 3 years and change in BMI z-scores from birth to age 3 years were significantly associated with components of fat-free mass and FM at age 11 years. Thus, our results are consistent with those of previous studies^14^^,^^16^ and suggest that Japanese health examinations of 3-year-old children,^20^ which are routinely performed for all children by local governments in Japan, are an effective tool for predicting body composition in later life.

The early programming hypothesis suggests a potential mechanism for the relationship between BMI in early childhood and body composition in later childhood.^29^ According to this hypothesis, hormonal programming induced by early nutritional experience and growth rate influences subsequent metabolism and hence growth rate.^9^^,^^30^ A recent study reported that high weight gain in infancy influenced both body fat and concentrations of ghrelin and adiponectin in adolescence.^31^ However, little evidence exists for this hypothesis, and mechanisms that might link early-life BMI and later body composition have not been identified.

The present study used height-normalized indices but did not express body composition as a percentage of body weight. VanItallie et al reported that expressing fat-free mass and FM as percentages of body weight is problematic. For instance, tall patients with protein-energy malnutrition can have fat-free mass and FM values similar to those of shorter, well-nourished individuals.^22^ In the present study, although BMI at age 3 years was positively associated with BMCI and FFSTMI, BMI at age 3 was inversely associated with later BMC/body weight (%BMC) and FFSTM/body weight (%FFSTM) (*r* = −0.159 and −0.287, respectively) in boys. The underlying reason for this heterogeneity might be that body weight at age 3 is positively associated with body weight at age 11. Thus, an increase in body weight at age 11 is also associated with lower %BMC and %FFSTM. We used height-normalized indices to allow for a clear interpretation of the relationship between BMI at age 3 and later body composition.

This study has several limitations worth noting. First, data were obtained from only 1 prefecture in Japan rather than by selection from the entire country. In addition, we have no data on the demographic characteristics of children who did not participate in the study (24.2% of the source population). However, mean body height and mean body weight of the 3-year-olds included in this study are consistent with those reported in a Japanese national survey. Standard growth charts for height and weight of Japanese children show that mean height is 93.3 cm for boys and 92.1 cm for girls, and mean weight is 13.8 kg for boys and 13.1 kg for girls at age 3.^32^ Height and weight at birth and age 11 were also similar to those in the national survey.^32^ As compared with a multicenter design, the present single-center study design is better for quality control of body composition measurements. A second limitation was that the study design was retrospective, and we did not confirm whether parents actually referred to information in the MCH Handbook. Therefore, the accuracy of anthropometric data at birth and age 3 is unclear. However, mean anthropometric values in the present study are consistent with those in the national survey, which supports the validity of completing questionnaires using information from MCH Handbooks. Third, we could not obtain a precise age in months for the 3-year-olds (eg, 3 years 0 months or 3 years 11 months). Children gain significant height and weight from 3 years 0 months to 3 years 11 months, and these gains might be relevant in classifying 3-year-olds as under- or overweight. However, change in BMI in both sexes was less than 0.2 kg/m^2^ from 3 years 0 months to 3 years 11 months in the Japanese national survey.^32^ Thus, in our view, this limitation does not affect the strength of predictions regarding body composition at age 11 years. Fourth, we did not conduct a validation study on self-reported maternal height and weight. However, it was reported that self-reported values for height and weight appear to be valid in Japanese adults.^33^ Fifth, although body composition in children who reach puberty is likely to be affected by hormonal activity, we could not ascertain pubertal status in this study. Thus, we could not control for the effect of puberty on body composition.

In conclusion, BMI at age 3 years was positively associated with BMC, FFSTM, trunk FM, and whole-body FM at age 11 years in Japanese children. Body weight in early childhood predicted pubertal body composition. Specifically, components of body composition in later life were significantly lower among children who were underweight in early childhood and significantly higher among children who were overweight in early childhood, as compared with those of normal-weight children.

## ONLINE ONLY MATERIALS

Abstract in Japanese.
