# Supplementary material for: Relationship Between Body Mass Index at Age 3 Years and Body Composition at Age 11 Years Among Japanese Children: The Shizuoka Population-Based Study
Source: J Epidemiol. 2012 Sep 5;22(5):411–6. doi: 10.2188/jea.JE20110113 (PMC3798635; doi:10.2188/jea.JE20110113)
Supplement: Abstract in Japanese. [file je-22-411-s001.pdf]

日本人における3歳時の体格と11歳時の体組成の関係：静岡ポピュレーションベース研究

甲田勝康[1]、中村晴信[2]、藤田裕規[1]、伊木雅之[1]

[1]近畿大学医学部公衆衛生学教室、[2]神戸大学大学院人間発達環境学研究科健康発達論

【背景】東アジアの集団において、幼児期の体格とその後の体組成の関連についての詳細な研究はない。今回は日本人集団における3歳時の体格と11歳時の体組成の関連について、二重エネルギーエックス線吸収法（DXA法）を用いて検討した。
【方法】調査対象集団は2008年から2010年に静岡県下の3つの公立小学校に在籍した726名の児童である。体組成はDXA法で測定し、幼児期の体格（BMI）は母子健康手帳から入手した。統計解析には一般線形モデルを用いた。
【結果】調査対象集団のうち550名から体組成と幼児期の体格のデータを得た。3歳時のBMIは11歳時の全身骨塩量（BMC）、全身除脂肪軟部組織量（FFSTM）、全身脂肪量（FM）と正の関連を示した。出生から3歳のBMI z-scoreの変化も11歳時のBMC、FFSTM、FMと正の関連を示した。交絡因子調整後の11歳時のBMC、FFSTM、FMの平均値は、共に3歳時に低体重であった者が正常体重よりも低く、3歳時に過体重であった者は正常体重よりも高かった。
【結論】3歳時の体格はその後の体組成を予測した。

キーワード：栄養、小児、体組成、発達
